# A limiting Current Oxygen Sensor Constituted of (CeO_2_)_0.95_(Y_2_O_3_)_0.05_ as Solid Electrolyte Layer and (CeO_2_)_0.75_(ZrO_2_)_0.25_ as Dense Diffusion Barrier Layer

**DOI:** 10.3390/s19163511

**Published:** 2019-08-10

**Authors:** Xiangnan Wang, Tao Liu, Jingkun Yu

**Affiliations:** School of Metallurgy, Northeastern University, Shenyang 110819, China

**Keywords:** limiting current oxygen sensor, YDC solid electrolyte, ZDC dense diffusion barrier, water vapor pressure

## Abstract

Using the co-precipitation method to synthesize (CeO_2_)_0.95_(Y_2_O_3_)_0.05_ (YDC) and solid reaction method to synthesize (CeO_2_)_0.75_(ZrO_2_)_0.25_ (ZDC), and the crystal structure, micro-structure, total conductivity and electronic conductivity of the two materials was measured with X-ray diffraction (XRD), scanning electron microscope (SEM), DC van der Pauw and Hebb-Wagner methods. A limiting current oxygen sensor was prepared with YDC solid electrolyte and a ZDC dense diffusion barrier layer by employing platinum pasting bonding. Sensing characteristics of the sensor were obtained at different conditions, including temperature (*T*), oxygen concentration (*x*(O_2_)) and water vapor pressure (*p*(H_2_O)), and the influence of various conditions on sensing performance was studied. The long-term stability of the sensor was measured in an oxygen concentration of 1.2% and at a temperature of 800 °C for 120 h. XRD results show that the phase structure of both YDC and ZDC belongs to the cubic phase. SEM results show that both YDC and ZDC layers are dense layers, which are then qualified to be the composition materials of the sensor. The limiting current (*I*_L_) of the sensor is obtained and the sensor exhibits good sensing characteristics to satisfy the Knudsen model. Log(*I*_L_·*T*) depends linearly on 1000/*T* with a squared correlation coefficient (*R*^2^) of 0.9904; *I*_L_ depends linearly on *x*(O_2_) with an *R*^2^ of 0.9726; and sensing characteristics are not affected by *p*(H_2_O). It was found that the oxygen sensor has good long-term stability.

## 1. Introduction

Electrochemical oxygen sensors are intended for oxygen concentration detection and are very essential to monitor and control the air fuel ratio (A/F) in industrial boilers and in automotive and metallurgical industries, which contributes a lot to the combustion process optimization, energy savings and emission reductions [1,2,3]. Concentration potential oxygen sensor has been widely studied in the past. It has high sensitivity near the theoretical air–fuel ratio and has been widely used. However, since the potential of the sensor is linearly related to the logarithm of the oxygen partial pressure, the sensor is less sensitive to oxygen concentration outside the theoretical air–fuel ratio accessory [4,5]. After the application of direct injection lean fuel combustion technology, it is necessary to measure oxygen concentration in a wide range [6,7]. There is a linear relationship between the limiting current of oxygen sensors and oxygen concentration in a wide range of oxygen concentrations. At the same time, the sensitivity of the sensors is high. Therefore, the limiting current oxygen sensor has successfully attracted public attention [8,9,10]. The limiting current oxygen sensor includes two types: pore type and dense type, according to different types of the diffusion barrier. The pores of the pore type sensor may be blocked by solid particles from the environment, resulting in interference of the sensing characteristics. Such a disadvantage of the pore type sensor has driven the dense type to become a hot research focus [11,12]. Scholars from both domestic China and foreign countries have conducted many researches and studies on limiting current oxygen sensors. Garzon et al. prepared a limiting current oxygen sensor constituted of 8 mol% Y_2_O_3_ stabilized ZrO_2_ (8YSZ) solid electrolyte and La_0.84_Sr_0.16_MnO_3_ (LSM) dense diffusion barrier. The results show that LSM is prone to chemical reaction with YSZ and cracking [13]. Peng et al. prepared a limiting current oxygen sensor with YSZ solid electrolyte and Pt/YSZ mixed materials diffusion barrier. The results show that the limiting current can achieve a plateau when the oxygen concentration range is from 0 to 1.8%, but the application of precious metal Pt greatly increases the cost [14]. Gao et al. prepared a limiting current oxygen sensor with La_0.8_Sr_0.2_Ga_0.8_Mg_0.2_O_3-*δ*_ (LSGM) as both a solid electrolyte and dense diffusion barrier. The results show that the limiting current plateau is obtained in the oxygen concentration range of 1.92%–21%, which shows good sensing characteristics. However, due to the high cost of raw materials, the complex preparation process of synthetic materials and the large use of platinum wire leads to the high cost [15]. Therefore, cracks do not easily appear between the solid electrolyte and diffusion barrier; the chemical stability is good at high temperature, and the cost controls of the sensor are important indicators. The doping of Y_2_O_3_ in CeO_2_ will increase the oxygen ionic conductivity of the material and become a solid electrolyte with good mechanical properties [16]. Meanwhile, the conductivity of ZrO_2_-doped CeO_2_ indicates that it has electronic conductivity and can be seen as a dense diffusion barrier [17,18]. Furthermore, the prices of raw materials are low and the synthetic process of synthetic materials is simple, which is conducive to industrial production. So, a limiting current oxygen sensor was prepared with YDC solid electrolyte and ZDC dense diffusion barrier, and the sensor may become a promising material for oxygen sensors due to the good chemical stability and high electrical conductivity.

In this paper, the researchers used co-precipitation method to synthesize YDC and solid reaction method to synthesize ZDC; and the characterization for the crystal structure, micro-structure, total conductivity, and electronic conductivity of the two materials was conducted. A limiting current oxygen sensor was prepared with YDC solid electrolyte and ZDC dense diffusion barrier by employing platinum pasting bonding. The impacts of temperature, oxygen concentration as well as water vapor pressure on the sensing characteristics of the oxygen sensor were studied, and the long-term stability of the oxygen sensor was also conducted.

## 2. Experimental

(CeO_2_)_0.95_(Y_2_O_3_)_0.05_ (YDC) was synthesized by co-precipitation method with analytical reagents Ce(NO_3_)_3_·6H_2_O (purity 99.95%), Y(NO_3_)_3_·6H_2_O (purity 99.99%) and NH_3_·H_2_O (purity 0.1 M) without prior purification treatment. (CeO_2_)_0.75_(ZrO_2_)_0.25_ (ZDC) was synthesized by solid reaction method with analytical reagents Ce_2_(CO_3_)_3_·*x*H_2_O (purity 99.99%), ZrOCl_2_·8H_2_O (purity 99.9%) and H_2_C_2_O_4_·2H_2_O (purity 99.99%) without prior purification treatment. The above two synthetic methods have a low synthesis temperature, which is conducive to energy saving and emission reduction. Firstly, weigh reagents Ce(NO_3_)_3_·6H_2_O and Y(NO_3_)_3_·6H_2_O, and dissolve the reagents in distilled water by intense agitation. Drip reagent NH_3_·H_2_O into nitrate salt solution and continuously stir the mixture until a pH of 9 is reached. Wash the obtained precipitate with distilled water and ethanol, then dry it at 70 °C, and calcine at 800 °C for 2 h to obtain YDC solid solution powder. Secondly, weigh the stoichiometric reagents Ce_2_(CO_3_)_3_·*x*H_2_O, ZrOCl_2_·8H_2_O and H_2_C_2_O_4_·2H_2_O, and mill them with agate beads for 96 h. Add 7 wt% Tween 60 into the above reagents at the 48^th^ h. Calcine at 600 °C for 4 h to obtain ZDC solid solution powder. Analyze the crystal structure of YDC and ZDC powders by XRD technology (Philips PW3040/60, Amsterdam, Netherlands). Press the YDC and ZDC powders and sinter them at 1600 °C for 6 h in air to obtain sintered bodies for testing. Characterize the micro-structure of sintered bodies YDC and ZDC by SEM equipment (Zeiss, Jena, Germany). The total conductivity and electronic conductivity of ZDC were measured by DC van der Pauw and Hebb-Wagner method with electrochemical workstation (LK98BII, Beijing, China) according to Reference [4]. Ionic conductivity was obtained by subtracting electronic conductivity from total conductivity.

**Figure 1:**
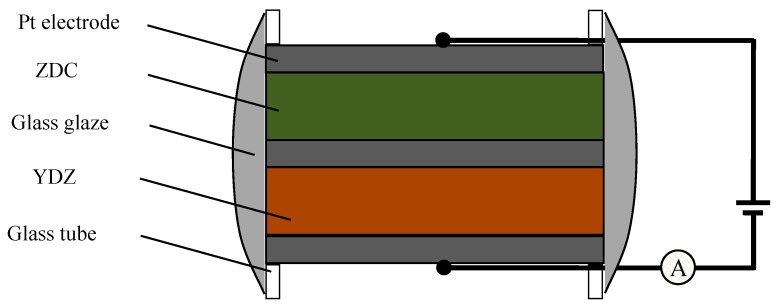
Structural drawing of the O_2_ sensor.

**Figure 2:**
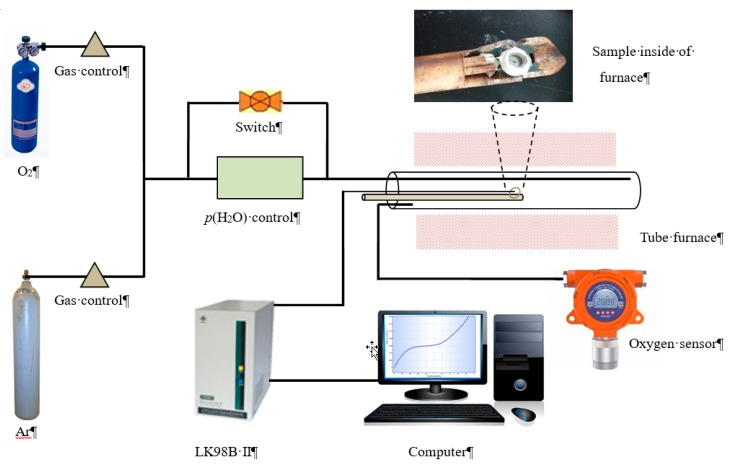
*I*-*V* characteristics testing system.

Prepare a limiting current oxygen sensor with YDC solid electrolyte (diameter 9.28 mm and thickness 0.86 mm) and ZDC dense diffusion barrier (diameter 8.40 mm and thickness 1.60 mm) by employing platinum pasting bonding, as shown in Figure 1. Test and record the current-voltage (*I*-*V*) characteristics of the oxygen sensor by electrochemical station (LK98BII, China) under different temperatures *T*, oxygen concentrations *x*(O_2_) and water vapor pressures *p*(H_2_O). Adjust the oxygen concentration by mixing different portions of argon and oxygen to get different Ar/O_2_ ratios. Pass dry Ar/O_2_ through LiCl·H_2_O saturated solution to adjust *p*(H_2_O) according to References [19,20,21]. Long-term stability of the sensor was measured in oxygen concentration of 1.2% and at temperature of 800 °C for 120 h. The total flow rate of Ar/O_2_ flows was about 100 mL·min^−1^. Figure 2 demonstrates the *I*-*V* characteristics testing system according to Reference [18].

## 3. Results and Discussion

### 3.1. YDC and ZDC

**Figure 3:**
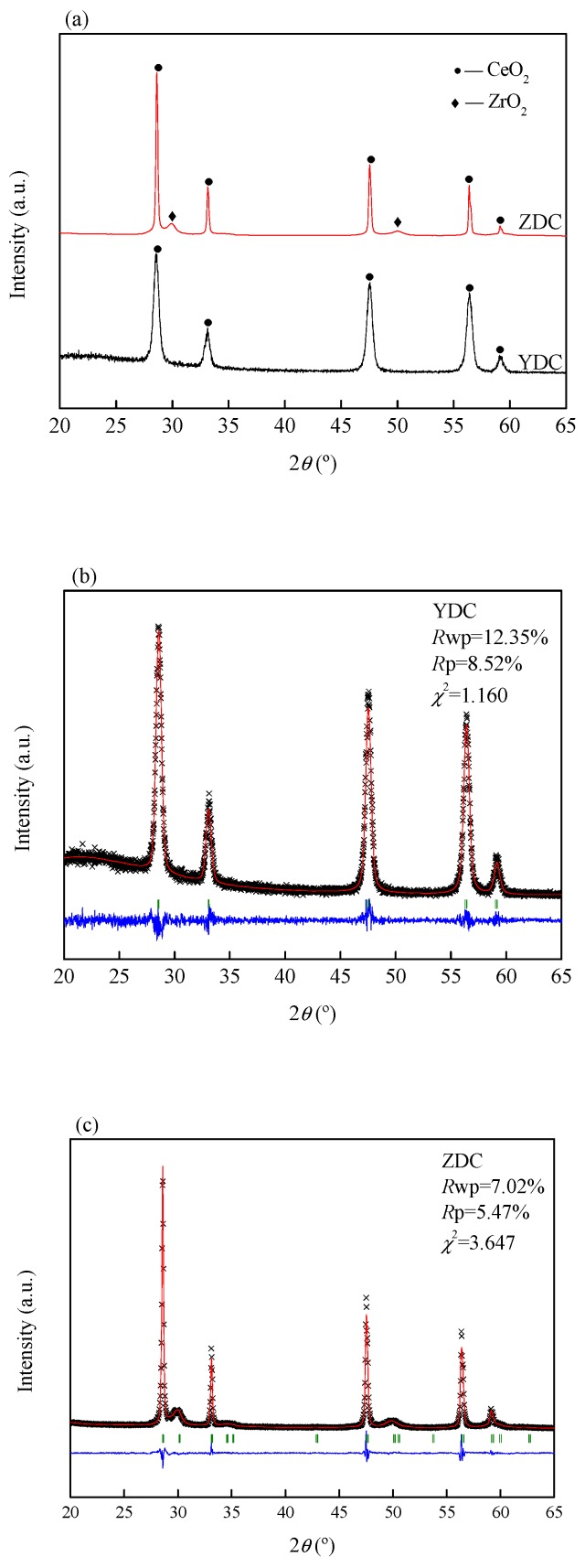
(**a**) Crystal structure of the YDC and ZDC, (**b**,**c**) unit cell constants of YDC and ZDC.

The crystal structure of the YDC and ZDC powders is shown in Figure 3a. It can be seen that YDC and ZDC are cubic phase CeO_2_, and a small amount of ZrO_2_ diffraction peaks are found in ZDC. The unit cell constants of YDC and ZDC were calculated by GSAS software refinement, as shown in Figure 3b,c [22]. The unit cell constant of YDC is *a* = *b* = *c* of 5.416 Å and the unit cell volume of 158.841 Å^3^. The unit cell constant of ZDC is *a* = *b* = *c* of 5.404 Å and the unit cell volume of 157.783 Å^3^. The unit cell constant and unit cell volume of YDC are larger than that of ZDC due to the Y ion radius being larger than the Zr ion radius.

**Figure 4:**
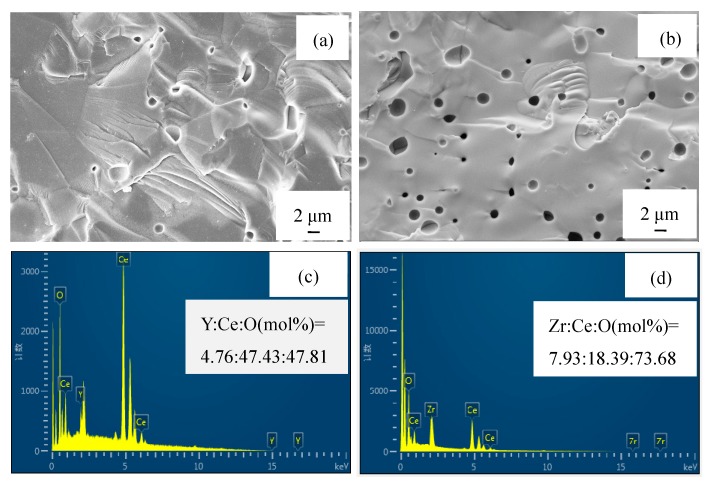
(**a**,**b**) SEM images of YDC and ZDC sintered bodies, (**c**,**d**) elemental distributions of YDC and ZDC.

Figure 4a,b shows the SEM images of YDC and ZDC sintered bodies from the cross-section side, and indicates that the cross-section grain of the sintered body is not obvious and the fracture is transgranular fracture. Figure 4c,d illustrates that the elemental distributions of YDC and ZDC, which also clearly show that the molar ratios of Y to Ce and Zr to Ce substantially comply with the stoichiometric ratios of YDC and ZDC solid solutions, respectively.

**Figure 5:**
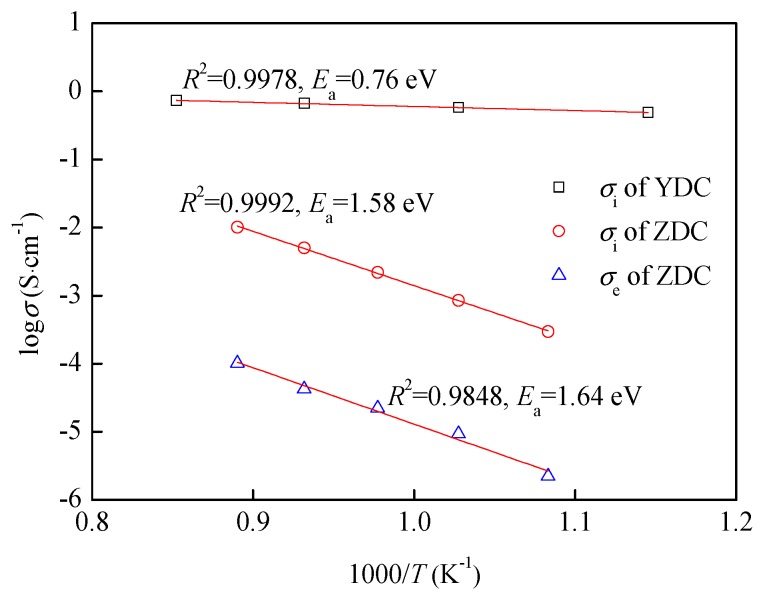
Ionic conductivity of YDC and ionic, electronic conductivity of ZDC.

The ionic conductivity of YDC and ionic, electronic conductivity of ZDC are shown in Figure 5 [16]. The electrical conductivities and temperature satisfy the Arrhenius law with an *R*^2^ of 0.9978 for ionic conductivity of YDC, *R*^2^ of 0.9992 for ionic conductivity of ZDC, and *R*^2^ of 0.9848 for electronic conductivity of ZDC, respectively. The electrical conductivities of YDC and ZDC meet the requirements of the solid electrolyte layer and dense diffusion barrier layer of the limiting current oxygen sensor, respectively.

### 3.2. I-V and T

**Figure 6:**
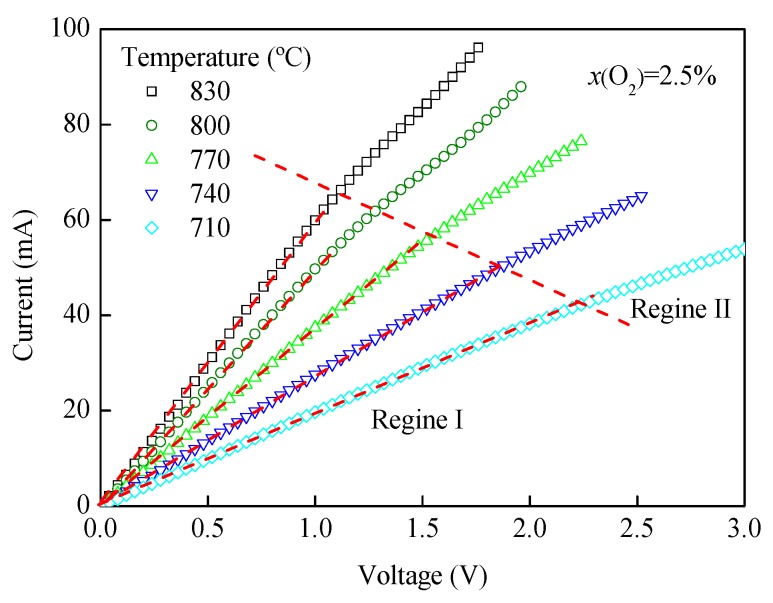
*I-V* characteristic curves at different temperatures.

The sensing characteristics of the oxygen sensor were obtained under the conditions of a temperature range of 710–830 °C and an oxygen concentration of 2.50%, as shown in Figure 6. As can be seen in the figure, the curves mainly contain two areas, namely the ohmic area and the limiting current plateau area. In area I, the output current increases linearly with the increase of applied voltage, owing to the ohmic behavior of the YDC layer of the sensor [23]. The ohmic slope has a relationship with the electrical conductivity of the solid electrolyte. The ohmic slope increases with increasing temperature due to an increase in the conductivity of the solid electrolyte with an increasing temperature. In area II, the slope of the *I-V* curve changes compared to that in area I, and the current in area II reaches plateau or close to plateau. Average current in area II is the limiting current value (I*_L_*) of the oxygen sensor. If there is no diffusion barrier, output current in area II will increase as the voltage increases, and the curve slope will be the same as that in area I. In fact, oxygen will be blocked by the dense diffusion barrier. The oxygen volume through the solid electrolyte layer will decrease; a new *I-V* slope will be generated in area II. The voltage at the junction of the two areas is the initial voltage of the oxygen sensor, and normally the lower the initial voltage value is, the more sensitive the sensor is. There is a negative correlation between initial voltage and temperature, meaning initial voltage will decrease as temperature increases, because the conductivity of the ZDC dense diffusion barrier will increase as temperature increases.

At present, the limiting current oxygen sensor with the La_0.8_Sr_0.2_Ga_0.8_Mg_0.2_O_3-*δ*_ (LSGM)-based solid electrolyte and dense diffusion barrier layer has the best sensing characteristics [10,15,24]. The oxygen ion conductivity of LSGM solid electrolyte is high, reaching 0.178 S·cm^−1^ at 800 °C [15]; the ion conductivity and electronic conductivity of the transition metal oxide-doped LSGM dense diffusion barrier are high [10,24]. The oxygen sensor can obtain a good limiting current plateau at different temperatures. The limiting current plateau tends to be apparent as the temperature increases, which is due to the increase of oxygen ion conductivity at high temperatures.

### 3.3. I-V and x(O_2_)

**Figure 7:**
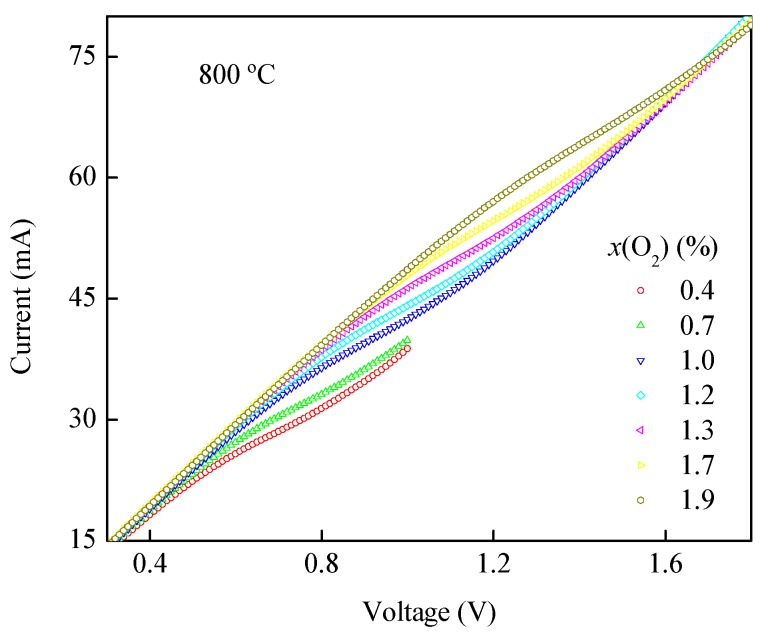
*I*-*V* characteristic curves in different *x*(O_2_).

Figure 7 demonstrates the *I*-*V* characteristic curves of the oxygen sensor in an oxygen concentration from 0.4% to 1.9% at 800 °C. When working, oxygen is adsorbed to the outside surface of the dense diffusion barrier, then, by absorbing two electrons at the (ZDC/Pt/air) triple phase boundaries at high temperatures, the absorbed oxygen becomes oxygen ions. Since the diffusion barrier is a mixed ionic-electronic conductor, the potential applied to both sides thereof is 0. Oxygen ions are transported from the surface of the ZDC layer to the ZDC/YDC interfaces under the driving of the oxygen pressure difference. Similarly, oxygen ions become oxygen molecules by losing electrons, and the oxygen molecules will be released at the triple-phase boundaries. The migration rate of oxygen ions from ZDC/YDC interfaces to YDC outside surfaces is affected by the voltage applied to the YDC layer. The oxygen pumping rate increases as the applied voltage increases, and the limiting current plateau can be obtained when the voltage increases to a certain value and the oxygen pumping rate from the YDC layer is limited by the oxygen diffusion rate from the ZDC layer. When the pumping rate of the YDC solid electrolyte is limited by the oxygen diffusion rate of the ZDC dense diffusion barrier and the voltage increases to a certain value, the limiting current plateau is obtained. The initial voltage is positively related to oxygen concentration, which is consistent with phenomena of other studies [15,24].

Similarly, the LSGM-based dense diffusion barrier layer limiting current oxygen sensor has a wide range of oxygen concentration detection. In Reference [10], the oxygen measurement range of the Fe_2_O_3_-doped LSGM dense diffusion barrier oxygen sensor is 0.2–20.9% at 800 °C. In Reference [24], a limiting current oxygen sensor with a Cr_2_O_3_-doped LSGM dense diffusion barrier has an oxygen concentration range of 0.88–7.23% at 700 °C, 0.88–10.94% at 750 °C and 1.92–21% at 800 °C, respectively. With the increase of temperature, the range of oxygen measurement increases. However, the raw materials used in LSGM synthesis are expensive, especially SrO and Ga_2_O_3_ (the price of SrO (AR) is 507 dollars per 100 g and the price of GaO (AR) is 231 dollars per 100 g). The synthesis of LSGM solid solution requires two-step synthesis, which is complicated by repeated grinding and high temperature calcination at 1450 °C. Therefore, it is necessary to continue to explore materials and processes suitable for industrial production of sensors. The material in this paper is low in price, simple in synthesis and low in calcination temperature. Although the oxygen measurement range is narrow, the sensitivity is high.

**Figure 8:**
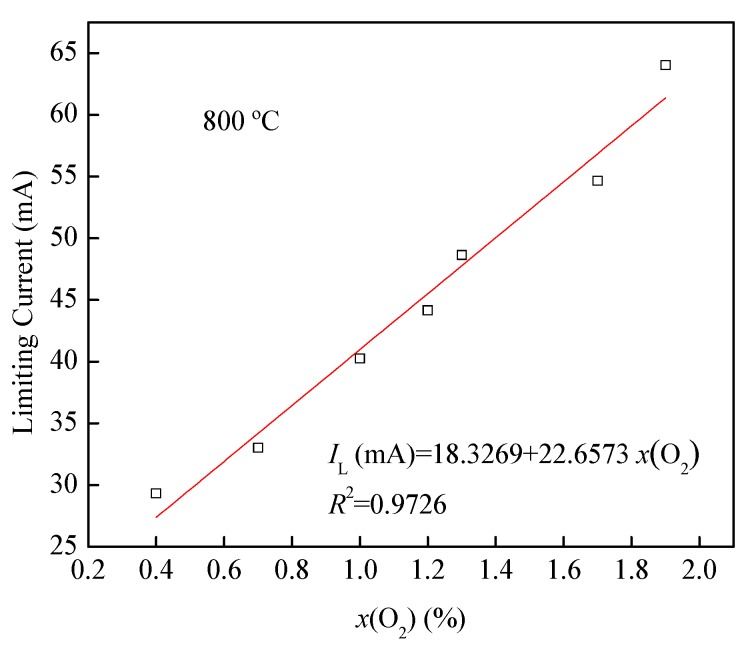
Limiting current and the oxygen concentration at the temperature of 800 °C.

Figure 8 demonstrates the linear correlation between the limiting current and the oxygen concentration at the temperature of 800 °C with *R*^2^ of 0.9726. Refer to Reference [2] for Knudsen diffusion correlation theory, which is the same as this correlation.

(1) $$I_{L}=\frac{4FD_{K}SP}{RTL}\cdot x(O_{2})$$

where $D_{K}$ is the oxygen diffusion coefficient, *P* the partial pressure difference between electrodes, *T* the temperature, *F* the Faraday constant, *R* the gas constant, *S* the total cross-sectional area, and *L* the length of the diffusion path, respectively.

Solid state theory of solid ion diffusion mode, diffusion coefficient ($D_{T}$) and temperature (*T*) is as follows:

(2) $$D_{T}=D_{O}\cdot \exp\left(-\frac{\varepsilon }{k_{B}T}\right)$$

where $D_{O}$ is the constant of the frequency factor, ε the activation energy for the diffusion process and $k_{B}$ the Boltzmann constant, respectively.

Equation (3) is obtained by simultaneous Equations (1) and (2):

(3) $$I_{L}=\frac{4FD_{O}SP}{RLT}\cdot x(O_{2})\cdot \exp(-\varepsilon /k_{B}T)$$

Assuming the oxygen concentration is stable, then:

(4) $$a=\frac{4FD_{O}SP}{RL}x(O_{2})$$

Equation (5) is obtained by introducing Equation (4) into Equation (3):

(5) $$I_{L}=a\cdot \frac{1}{T}\cdot \exp(-\varepsilon /k_{B}T)$$

Equation (6) is obtained by solving the logarithm of Equation (5):

(6) $$\log I_{L}=A-\log T-\frac{\varepsilon }{k_{B}T}$$

and then,

(7) $$B=-\frac{\varepsilon }{k_{B}}$$

So we get Equation (8):

(8) $$\log I_{L}=A-\log T+B\cdot \frac{1}{T}$$

Equation (9) is obtained by sorting out:

(9) $$\log(I_{L}\cdot T)=A+B\cdot \frac{1}{T}$$

**Figure 9:**
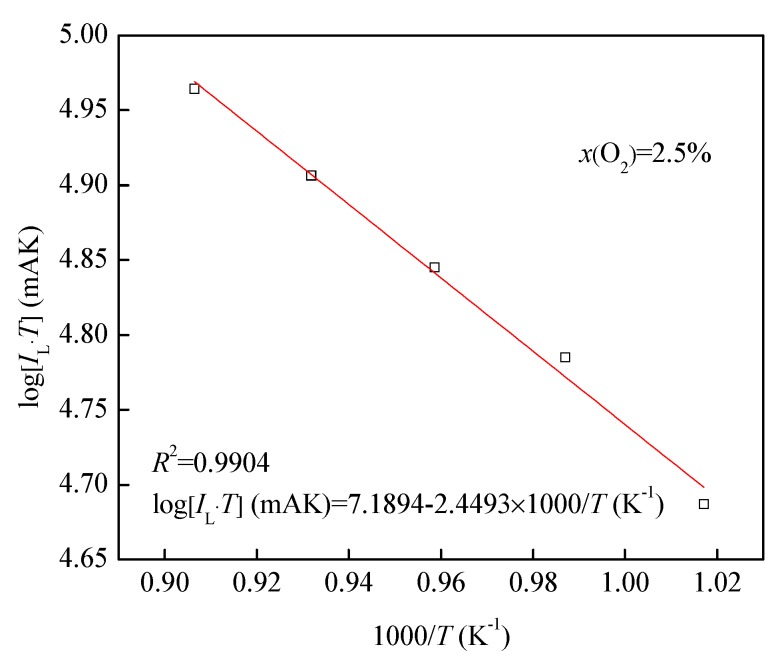
The $\log(I_{L}\cdot T)$ and 1000/*T* in *x*(O_2_) of 2.5%.

Figure 9 demonstrates the linear relationship between $\log(I_{L}\cdot T)$ and 1000/*T* in *x*(O_2_) of 2.5% according to Equation (9) with an *R*^2^ of 0.9904. This relationship meets the Knudsen diffusion model.

### 3.4. I-V and p(H_2_O)

**Figure 10:**
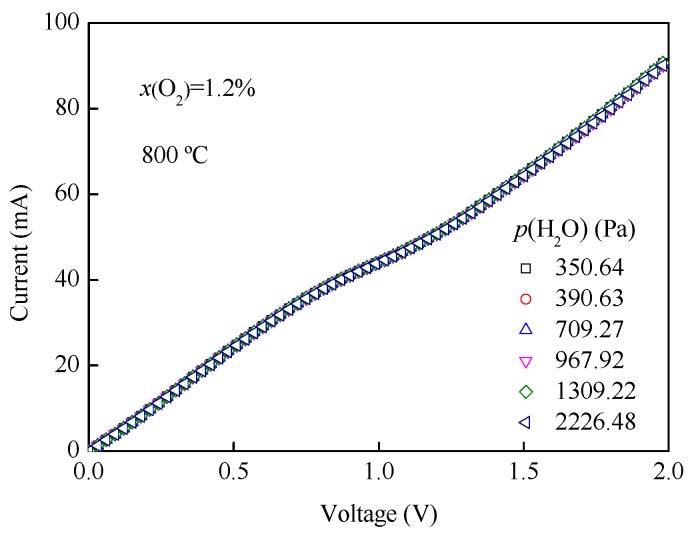
*I-V* characteristic curves and *p*(H_2_O) in an oxygen concentration of 1.2% and at a temperature of 800 °C.

Figure 10 demonstrates the effect of *p*(H_2_O) on *I-V* characteristic curves in an oxygen concentration of 1.2% and at a temperature of 800 °C. As can be seen, the sensing characteristics of the oxygen sensor obtained almost coincide under different *p*(H_2_O), indicating that the *p*(H_2_O) does not have significant effect on the *I*-*V* characteristics within the test range. Some studies about the influence of *p*(H_2_O) on the limiting current oxygen sensor show that the porous type sensor is affected by *p*(H_2_O), while the dense type sensor is not affected [18,25,26]. The dense type oxygen sensor has excellent selectivity to oxygen, which is an excellent indicator of the sensor.

So far, the LSGM-based dense diffusion barrier layer limiting current oxygen sensors with the best sensing performances has not studied the effects of water vapor pressure on the sensing performance of the sensor [10,15,24]. However, in my previous researches, such as in References [27,28], the sensing performance of the dense diffusion barrier limiting current oxygen sensor is not affected by water vapor pressure, because the dense diffusion barrier is oxygen–ion conductive.

### 3.5. Long-Term Stability

**Figure 11:**
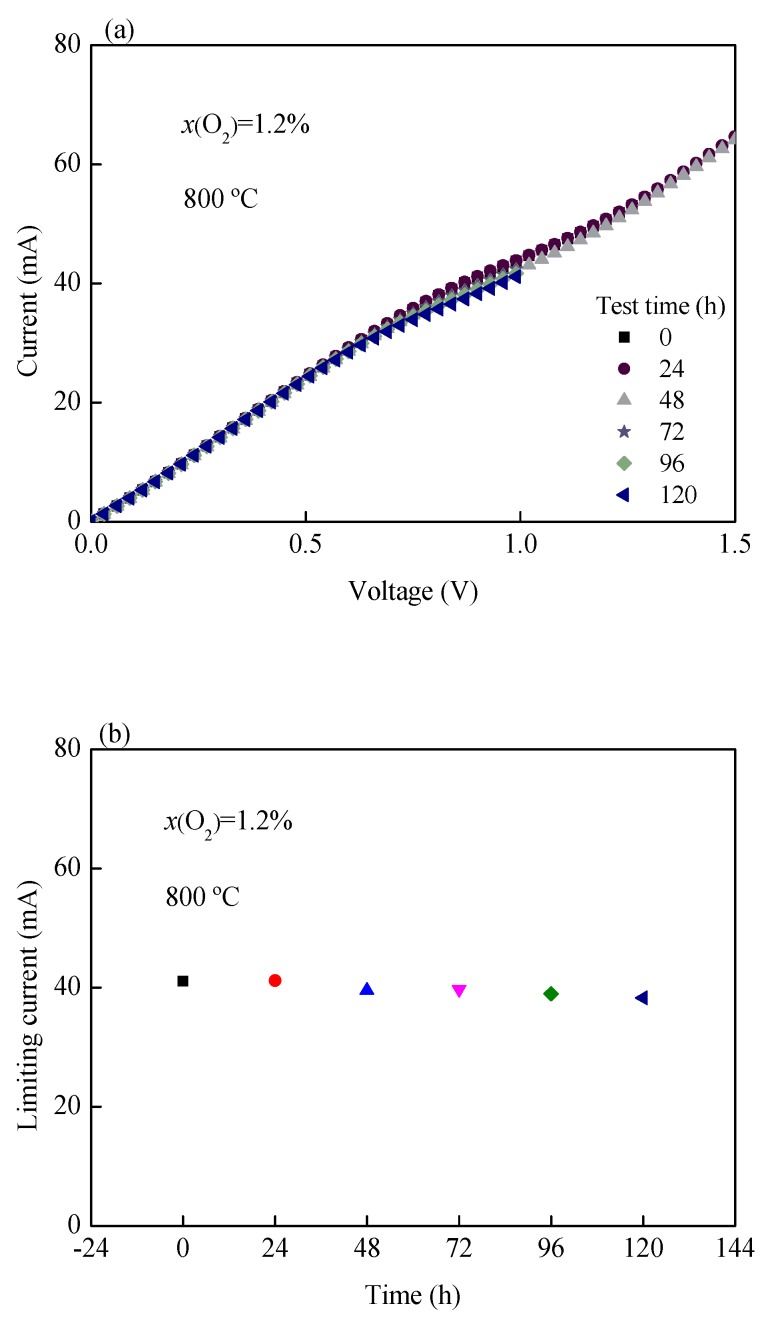
(**a**) *I-V* curves from 0 to 120 h, (**b**) limiting current from 0 to 120 h.

The sensing performance of the limiting current oxygen sensor was tested in an oxygen concentration of 1.2% and at a temperature of 800 °C for 120 h, as shown in Figure 11. Figure 11a represents the *I-V* characteristic curves of the oxygen sensor obtained at different times. It can be found that the limiting current plateau of the oxygen sensor decreases very little. The limiting current of the oxygen sensor was plotted with the test time, as shown in Figure 11b. It is found that the limiting current value decreases slightly. By comparison, we find that the limiting current value of the oxygen sensor in Reference [27] decreases with the increase of test time, which may be due to the increase of polarization resistance. Furthermore, in Reference [27], the applied voltage is 0–2.5 V, while in this paper, it is 0–1.5 V. The decrease of the applied voltage is beneficial to the service life of the oxygen sensor, which is also the reason for the smaller reduction of the limiting current. Further research is needed on the specific reasons for the reduction of the limiting current value. Long-term stability is an important index of the oxygen sensor, which is particularly important for industrial production and application.

## 4. Conclusions

Using the co-precipitation method to synthesize YDC and the solid reaction method to synthesize ZDC, we prepared a YDC solid electrolyte and ZDC dense diffusion barrier-based limiting current oxygen sensor by platinum pasting bonding method. XRD results show that YDC and ZDC belong to the cubic phase structure. SEM results show that the sintered bodies are dense and conform to the materials for limiting current oxygen sensor. The oxygen sensor has good sensing performance, which is as follows: log(*I*_L_·*T*) depends linearly on 1000/*T* with an *R*^2^ of 0.9904, *I*_L_ depends linearly on *x*(O_2_) with an *R*^2^ of 0.9726 and sensing characteristics are not affected by *p*(H_2_O), and the limiting current decreases very little with the increase of test time from 0 to 120 h.

## References

[1] Liu T., Zhang X.F., Yuan L., Yu J.K. (2015). A review of high-temperature electrochemical sensors based on stabilized zirconia. Solid State Ion..

[2] Han J.X., Zhou F., Bao J.X., Wang X.J., Song X.W. (2013). A high performance limiting current oxygen sensor with Ce_0.8_Sm_0.2_O_1.9_ electrolyte and La_0.8_Sr_0.2_Co_0.8_Fe_0.2_O_3_ diffusion barrier. Electrochim. Acta.

[3] Liu T., Zhang X.F., Wang X.N., Yu J.K., Li L. (2016). A review of zirconia-based solid electrolytes. Ionics.

[4] Wang X.N., Liu T., Yu J.K., Mo Y.C., Yi M.Y., Li J.Y., Li L. (2018). Preparation and electrical property of CaZr_0.7_M_0.3_O_3_ (M=Fe, Cr and Co) dense diffusion barrier for application in limiting current oxygen sensor. Sens. Actuator B Chem..

[5] Brailsford A.D., Logothetis E.M. (1998). Selected aspects of gas sensing. Sens. Actuator B Chem..

[6] Ivers-Tiffée E., Härdtl K.H., Menesklou W., Riegel J. (2001). Principles of solid state oxygen sensors for lean combustion gas control. Electrochim. Acta.

[7] Riegel J., Neumann H., Wiedenmann H.M. (2002). Exhaust gas sensors for automotive emission control. Solid State Ion..

[8] Xia C.Y., Lu X.C., Yan Y., Wang T.Z., Zhang Z.M. (2011). Simulation of the transient response of limiting current oxygen sensor. Sens. Actuator B Chem..

[9] Liu T., Gao X., He B.G., Yu J.K. (2016). A limiting current oxygen sensor based on LSGM as solid electrolyte and LSGMN (N = Fe, Co) as dense diffusion barrier. J. Mater. Eng. Perform..

[10] Zhang X.F., Liu T., Yu J.K., Gao X., Jin H.B., Wang X.N., Wang C. (2017). A limiting current oxygen sensor with La_0.8_Sr_0.2_(Ga_0.8_Mg_0.2_)_1-x_Fe_x_O_3-δ_ dense diffusion barrier. J. Solid State Electrochem..

[11] Liu T., Wang X.N., Zhang X.F., Gao X., Li L., Yu J.K., Yin X.T. (2018). A limiting current oxygen sensor prepared by a co-pressing and co-sintering technique. Sens. Actuator B Chem..

[12] Mo Y.C., Liu T., Wang C. (2019). A limiting current oxygen sensor based on (La_0.4_Ce_0.6_O_2-δ_)_0.96_(FeO_1.5_)_0.04_ as dense diffusion barrier. Ceram. Int..

[13] Garzon F., Raistrick I., Brosha E., Houlton R., Chung B.W. (1998). Dense diffusion barrier limiting current oxygen sensors. Sens. Actuator B Chem..

[14] Peng Z.Y., Liu M.L., Balko E. (2001). A new type of amperometric oxygen sensor based on a mixed-conducting composite membrane. Sens. Actuator B Chem..

[15] Gao X., Liu T., Yu J.K., Li L. (2017). Limiting current oxygen sensor based on La_0.8_Sr_0.2_Ga_0.8_Mg_0.2_O_3-δ_ as both dense diffusion barrier and solid electrolyte. Ceram. Int..

[16] Wang C.Z. (2000). Solid Electrolytes and Chemical Sensors.

[17] Wang X.N., Liu T., Wang C., Yu J.K., Li L. (2017). Crystal structure, microstructure, thermal expansion and electrical conductivity of CeO_2_-ZrO_2_ solid solution. Adv. Appl. Ceram..

[18] Wang X.N., Liu T., Yu J.K., Li L., Zhang X.F. (2019). A new application of Ce_x_Zr_1-x_O_2_ as dense diffusion barrier in limiting current oxygen sensor. Sens. Actuator B Chem..

[19] Gokcen N.A. (1951). Vapor pressure of water above saturated lithium chloride solution. J. Am. Chem. Soc..

[20] Chung T.W., Luo C.M. (1999). Vapor pressures of the aqueous desiccants. J. Chem. Eng. Data.

[21] Kolár P., Nakata H., Tsuboi A., Wang P., Anderko A. (2005). Measurement and modeling of vapor-liquid equilibria at high salt concentrations. Fluid Phase Equilib..

[22] Toby B.H. (2001). EXPGUI, a graphical user interface for GSAS. J. Appl. Cryst..

[23] Usui T., Asada A., Nakazawa M., Osanai H. (1989). Gas polarographic oxygen sensor using an oxygen / zirconia electrolyte. J. Electrochem. Soc..

[24] Gao X., Liu T., Zhang X.F., He B.G., Yu J.K. (2017). Properties of limiting current oxygen sensor with La_0.8_Sr_0.2_Ga_0.8_Mg_0.2_O_3-δ_ solid electrolyte and La_0.8_Sr_0.2_(Ga_0.8_Mg_0.2_)_1-x_Cr_x_O_3-δ_ dense diffusion barrier. Solid State Ion..

[25] Mari C.M., Rabotti G. (1999). Humidity determination by solid state limiting current sensor. Solid State Ion..

[26] Akasaka S. (2016). Thin film YSZ-based limiting current-type oxygen and humidity sensor on thermally oxidized silicon substrates. Sens. Actuator B Chem..

[27] Wang X.N., Liu T., Yu Y.K., Li L. (2018). The effect of Fe doping on the electrical conductivities of CaZrO_3_ and its sensing performance in limiting current oxygen sensor. J. Alloy. Compd..

[28] Wang X.N., Liu T., Yu Y.K. (2019). An application of (4YSZ)_0.93_(Fe_2_O_3_)_0.07_ in limiting current oxygen sensor. Sci. Rep..

